# Epidemiology, Mechanisms, and Diagnosis of Drug-Induced Anaphylaxis

**DOI:** 10.3389/fimmu.2017.00614

**Published:** 2017-05-29

**Authors:** Maria Isabel Montañez, Cristobalina Mayorga, Gador Bogas, Esther Barrionuevo, Ruben Fernandez-Santamaria, Angela Martin-Serrano, Jose Julio Laguna, Maria José Torres, Tahia Diana Fernandez, Inmaculada Doña

**Affiliations:** ^1^Research Laboratory, IBIMA–Regional University Hospital of Malaga–UMA, Málaga, Spain; ^2^Andalusian Center for Nanomedicine and Biotechnology—BIONAND, Málaga, Spain; ^3^Allergy Unit, IBIMA–Regional University Hospital of Malaga–UMA, Málaga, Spain; ^4^Allergy Unit, Cruz Roja Hospital, Madrid, Spain

**Keywords:** anaphylaxis, drugs, IgE, MAS-related G protein-coupled receptor, IgG, *in vivo* diagnosis, *in vitro* tests

## Abstract

Anaphylaxis is an acute, life-threatening, multisystem syndrome resulting from the sudden release of mediators by mast cells and basophils. Although anaphylaxis is often under-communicated and thus underestimated, its incidence appears to have risen over recent decades. Drugs are among the most common triggers in adults, being analgesics and antibiotics the most common causal agents. Anaphylaxis can be caused by immunologic or non-immunologic mechanisms. Immunologic anaphylaxis can be mediated by IgE-dependent or -independent pathways. The former involves activation of Th2 cells and the cross-linking of two or more specific IgE (sIgE) antibodies on the surface of mast cells or basophils. The IgE-independent mechanism can be mediated by IgG, involving the release of platelet-activating factor, and/or complement activation. Non-immunological anaphylaxis can occur through the direct stimulation of mast cell degranulation by some drugs, inducing histamine release and leading to anaphylactic symptoms. Work-up of a suspected drug-induced anaphylaxis should include clinical history; however, this can be unreliable, and skin tests should also be used if available and validated. Drug provocation testing is not recommended due to the risk of inducing a harmful reaction. *In vitro* testing can help to confirm anaphylaxis by analyzing the release of mediators such as tryptase or histamine by mast cells. When immunologic mechanisms are suspected, serum-sIgE quantification or the use of the basophil activation test can help confirm the culprit drug. In this review, we will discuss multiple aspects of drug-induced anaphylaxis, including epidemiology, mechanisms, and diagnosis.

## Introduction

Anaphylaxis is a severe, potentially life-threatening, generalized, or systemic hypersensitivity reaction that results from the sudden release of mediators derived from mast cells and basophils *via* degranulation (1–3). Drugs are the most common anaphylaxis triggers in adults (4–6), representing up to 10% of overall causes in outpatient studies (7), whereas for emergency department and hospitalized patients the proportion ranges from 27–60% (4, 8, 9).

While the symptoms of anaphylaxis can involve any organ, the most commonly affected are the cutaneous (affecting around 88% of cases), respiratory (76.1%), cardiovascular (41.9%), and gastrointestinal systems (12.8%) (10). Severe reactions (associated with hypotension) are more likely to be drug induced (4), representing up to 58% of fatal anaphylaxis (11).

Although anaphylaxis usually presents as an acute episode, mast cells can release mediators hours after the initial reaction causing a biphasic or late phase reaction. These biphasic and protracted cases can occur in up to 10% of drug-induced anaphylaxis instances (12).

In this paper, we will review the epidemiology, mechanisms, *in vivo* and *in vitro* diagnosis, and management of drug-induced anaphylaxis.

## Epidemiology of Drug-Induced Anaphylaxis

Estimates of the prevalence of anaphylaxis can vary, mainly due to a lack of consensus on the definition of anaphylaxis, the source of data, and populations evaluated. One study calculated an overall incidence of 3–50 per 100,000 person years and a lifetime prevalence of 0.05–2% (8). The incidence of drug-induced anaphylaxis has been estimated to range from 0.04 to 3.1% (13–15) and to be responsible for one case in every 4,000 emergency department visits (16), with a fatality rate of 0.65% (17). In terms of changes over time, drug-induced anaphylaxis has increased by 150% and mortality rates by 300% in parallel with an increasing incidence of overall anaphylaxis from 1997 to 2005 (4).

## Drugs Causing Anaphylaxis

Anaphylaxis can be induced by a range of drugs, being analgesics and antibiotics the most commonly involved, which may be partly explained by their frequent use in current medical practice (9, 10, 18).

### Non-Steroidal Anti-inflammatory Drugs (NSAIDs)

Non-Steroidal Anti-inflammatory Drugs are the most frequent triggers of drug-induced anaphylaxis, being responsible for 48.7–57.8% of incidents (10, 18). These are typically immunological reactions (19) that can be driven by an IgE-dependent mechanism with sufferers showing tolerance to other strong COX-1 inhibitors (19, 20). However, anaphylaxis induced by cross hypersensitivity to NSAIDs, driven by an IgE-independent mechanism, has also been described (21–23). The most common culprits are pyrazolones, propionic acid derivatives, diclofenac, and paracetamol (10, 19, 22, 24). The incidence of NSAID-induced anaphylaxis with concomitant asthma, rhinosinusitis, and nasal polyps ranges from 2%, in children, to 97%, in adults (25). The prevalence ranges from 0.06 to 0.9% (26), with acetyl salicylic acid accounting for approximately 3% of all instances of anaphylaxis (27).

### Beta-Lactam Antibiotics

Beta-lactams represent the second most frequent cause of drug-induced anaphylaxis, accounting for 14.3% of cases (18), with amoxicillin being the most common trigger (5). Recently, clavulanic acid, usually prescribed in combination with amoxicillin, has also been implicated (28, 29). Cases with cephalosporins, carbapenems, and monobactams are rare (30–32). The rate of anaphylactic reactions to beta-lactams has been estimated to be between 1 and 5 per 10,000 patient courses of treatment (33) and these drugs account for 75% of all fatal anaphylactic episodes in the US each year (34).

### Non-Beta-Lactam Antibiotics

Up to 75% of patients with immediate hypersensitivity to fluoroquinolones develop anaphylaxis, with moxifloxacin being the most common culprit, followed by ciprofloxacin (35). As a whole, fluoroquinolones are responsible for 9% of severe antibiotic anaphylaxis (31).

Anaphylaxis to sulfonamides, trimethoprim, and macrolides are rare (36, 37). Cases of vancomycin IgE-mediated anaphylaxis have been occasionally reported (38); however, this drug more commonly induces direct mast cell stimulation, associated with rapid intravenous administration, and characterized by flushing and pruritus, known as “red man syndrome” (24). In addition, this drug may lead to more severe reactions including hypotension and muscle spasms (24).

### Radiocontrast Media (RCM)

Reactions to RCM with systemic symptoms have decreased with the introduction of non-ionic, low osmolar agents, down from 12.1 to 0.04% of patients receiving RCM (39, 40). Although these reactions have historically been deemed non-IgE mediated, it should be noted that both ionic and non-ionic RCM may trigger IgE-mediated anaphylaxis (35, 41–43). Anaphylaxis to gadolinium agents is much less frequent with an incidence of 0.004–0.01% (44, 45). Older age and multiple previous exposures to RCM increase the risk of having anaphylaxis associated with hypotension. Fatalities have been reported even after the introduction of non-ionic RCM, with most cases lacking predictable risk factors (46). RCM accounted for 27% of fatal drug-induced anaphylaxis (11).

### Proton Pump Inhibitors (PPIs)

Anaphylaxis to PPIs is also becoming more common, representing 36–80% of all hypersensitivity reactions to these drugs (47–50). Lansoprazole is the most commonly involved agent (68.3–26.41%), followed by esomeprazole (30.18–10.0%), pantoprazole (20.0%), omeprazole (18.86–1.7%), and rabeprazole (6.7–3.77%) (51).

### Neuromuscular Blocking Agents (NMBAs)

Neuromuscular blocking agents are often considered one of the group of drugs that most frequently cause allergic reactions during the perioperative period (52–54). Reactions may be IgE mediated or due to the non-specific release of histamine (52). There are geographical differences and changes over time in the epidemiology of perioperative anaphylaxis. The incidence of intraoperative anaphylactic reactions has been estimated to be 1 in 1,250–10,000 anesthetics in France (54, 55), being lower in Australia and New Zealand (1 in 10,000–20,000) (56). Although mortality from perioperative anaphylaxis has been previously reported between 3 and 9% (54), a more recent study put it in the range of 0–1.4% (56). A study from France reported that for 59% of intraoperative anaphylactic reactions, the etiological agent was an NMBA, more specifically suxamethonium, vecuronium, pancuronium, alcuronium, atracurium, or gallamine (57). More recent studies report rocuronium and succinylcholine at higher risk of anaphylaxis, whereas pancuronium and cis-atracurium are reported to be the NMBAs associated with the lowest incidence of anaphylaxis (53, 58–62).

### Sugammadex

Sugammadex is a synthetic g-dextrin derivative designed to selectively bind to steroidal NMBAs. Cases of anaphylaxis to sugammadex have been recently reported (63–65) being an IgE-mediated mechanism suggested in several cases as patients gave positive skin tests and flow cytometry results (66, 67). It has been suggested that treatment of rocuronium-induced anaphylaxis should include the administration of sugammadex (68, 69). However, other studies have concluded that sugammadex is unlikely to modify the clinical course of an established allergic reaction (70).

### Hypnotics

Barbiturates induce frequent reactions due to the ability to elicit direct histamine release, although IgE-mediated anaphylaxis has also been described (71, 72). Reactions were also frequent with hypnotics using Cremophor EL as solubilizer; however, since propofol was formulated in soybean oil emulsion, the rate of reactions decreased (54, 73, 74). It has been suggested that allergic patients to eggs or soy should avoid propofol because of the presence of lecithins in the propofol vehicle; however, this has not been confirmed (75, 76) and currently is not recommended (77).

### Opioids

Hypersensitivity reactions to opioids are rare, and most cases are due to the non-immunologic induction of histamine release, being pruritus the most frequent symptom. Although rare, isolated episodes of IgE-mediated anaphylaxis to opioids have been described (78–80). The most common offenders inducing non-imnunologic reactions are the low-potency opiates (meperidine, codeine, and morphine); interestingly, high-potency opioids such as fentanyl and hydromorphone are less likely to cause histamine release (81).

### Chlorhexidine

Chlorhexidine is a skin antiseptic widely used in surgical settings. Perioperative anaphylaxis induced by chlorhexidine is quite frequent in UK or Denmark (82, 83) but rare in France maybe due to its limited use (84). Sensitization to chlorhexidine can occur from home products such as mouthwash, toothpaste, dressings, ointments, and over the counter disinfectant solutions (85).

### Dyes

Triarylmethane dyes, methylene blue, patent blue V, and isosulfan blue induce a relatively frequent rate of perioperative anaphylaxis due to their wide use in sentinel lymph node mapping in cancer surgery. Reactions may be induced by direct mast cell and/or basophil activation and specific IgE (sIgE) sensitization (86–88).

### Colloids

The incidence of anaphylaxis to colloids has been estimated to range from 0.033 to 0.22% (89). Gelatins and dextrans are more commonly associated with reactions than albumin and hetastarch (90).

## Factors Increasing the Risk of Drug-Induced Anaphylaxis

### Clinical Factors

Older age and intravenous administration have been shown to be associated with higher rates of drug-induced anaphylaxis (11) and an increased risk of severe reaction (91, 92). Other factors associated with the prevalence of fatal drug-induced anaphylaxis include race, with African-Americans being shown to have higher prevalence (11), the interruption of prior therapy creating gaps in administration (93) and decreased platelet-activating factor (PAF) acetylhydrolase activity (92). The role of atopy in predisposing an individual to drug-induced anaphylaxis is controversial (94) and underlying mast cell disease has not been described as a predisposing factor (95). Further research is needed to better identify patients at risk and to design preventive strategies to reduce the frequency of drug-induced anaphylaxis.

### Cofactors

The presence of several cofactors can increase the risk of suffering anaphylaxis and are reported to be relevant in up to 30% of anaphylaxis episodes (96). They include treatment with drugs such as NSAIDs, PPIs, or angiotensin-converting enzyme inhibitors; the presence of concomitant diseases (asthma, mastocytosis, and cardiovascular diseases); and other factors such as alcohol, emotional stress, or menstruation (96, 97).

## Anaphylaxis Mechanisms

**Figure 1:**
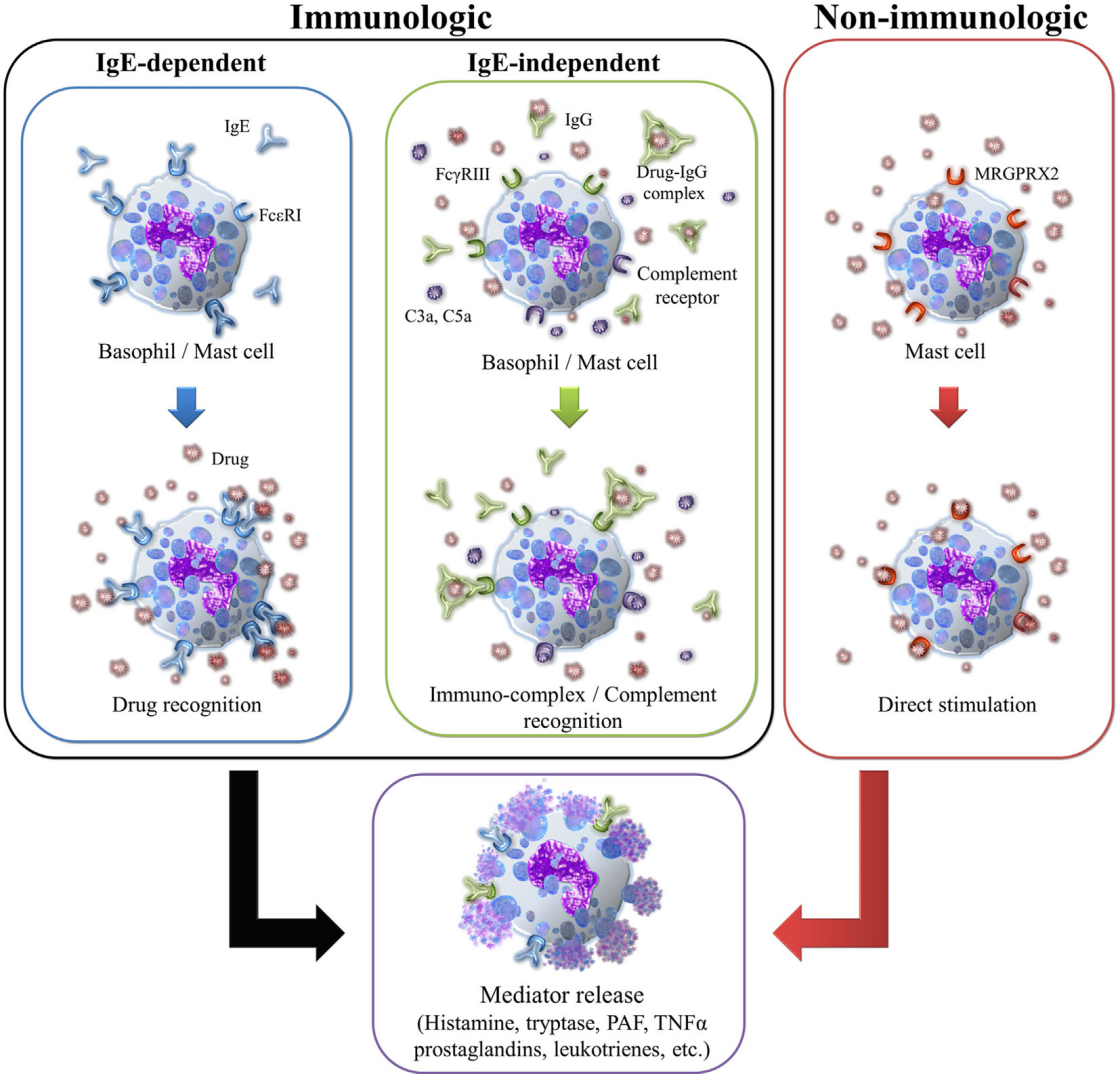
**Different mechanisms of mast cell or basophil activation induced by drugs**.

Anaphylaxis can be classified as immunologic and non-immunologic depending on the underlying mechanism; either type of reaction can be induced by drugs (98, 99). In some cases, the trigger cannot be identified; such reactions are classified as idiopathic anaphylaxis (100). Different mechanisms and pathways may be involved as illustrated in Figure 1. Immunologic anaphylaxis can be mediated by an IgE-dependent or -independent mechanism (101), whereas non-immunologic anaphylaxis involves direct mast cell activation (102–104). Independent of the underlying mechanism, allergic symptoms are similar and caused by the release of mediators such as histamine, tryptase, PAF, cysteinyl leukotrienes, and others (1). Histamine is responsible for flushing, pruritus, rhinorrhea, tachycardia, and bronchospasm *via* the induction of smooth muscle constriction and the increase of vascular permeability. Tryptase activates several pathways, including the complement cascade, coagulation pathway, and the kallikrein–kinin system, contributing to the development of hypotension and angioedema. PAF and cysteinyl leukotrienes also enhance vascular permeability and the development of hypotension (101).

### Immunologic Anaphylaxis

This can be induced by IgE-dependent or -independent mechanisms and mediated by the production of antibodies or the activation of the complement pathway (97).

*The IgE-dependent mechanism* or classical pathway involves a sensitization process including the activation of Th2 cells by the drug, inducing sIgE. This IgE binds to the FcεRI receptor on mast cells, basophils, or both. The cross-linking of two or more of these receptors by the hapten upon subsequent contact, initiates a complex intracellular signaling cascade that leads to degranulation and the release of preformed mediators such as histamine and tryptase. These cause the allergic symptoms and activate other inflammatory cells that can in turn release additional mediators and stimulate the production of others such as prostaglandin D2 and cysteinyl leukotrienes, which serve to amplify the allergic reaction. Two main mechanisms of degranulation have been recently proposed that may be related to reaction severity: piecemeal and anaphylactic degranulation (105). The former is associated with the upregulation of CD203c on basophils (106) by the formation of small vesicles from the histamine-containing granules, which are rapidly shuttled to the plasma membrane (107, 108). This process may be linked to stimulation by certain drugs and the development of more severe reactions like anaphylactic shock (105, 109). In the second mechanism, the main histamine-containing granules are fused to the plasma membrane, releasing the entire contents to the extracellular space and exposing CD63 on the surface of basophils (106). This second process is slower than piecemeal degranulation and could be related to the development of anaphylaxis (110). Penicillins and NMBA are considered the main triggers of IgE-mediated anaphylaxis induced by drugs (54, 111, 112).

*The IgE-independent mechanisms* can be mediated by IgG antibodies or by complement (97, 113). IgG-mediated anaphylaxis has been demonstrated in mouse models and involves the release of PAF by basophils, macrophages, or neutrophils after the interaction of the drug with specific IgG (sIgG) bound to FcγRIII. Although this mechanism has not been fully established in humans, some studies have shown that PAF is an essential mediator in anaphylaxis (92, 114). Biological agents have been shown to induce anaphylaxis without the presence of detectable sIgE but with high levels of sIgG, as occurs with patients transfused with IgA (115, 116), treated with infliximab or adalimimab (117, 118), and other biological factors (119–121). Complement activation can be induced through the presence of IgG immunocomplex, but also with drugs solubilized in therapeutic liposomes and lipid-based excipients under physiological conditions. This mechanism leads to the release of C3a, C5a, and C5b-9, which trigger activation of mast cells, basophils, and other cells *via* their specific receptors, causing degranulation and mediator release (97).

IgE-independent mechanism is clinically indistinguishable from IgE-mediated anaphylaxis. Among the most common causes of IgE-independent anaphylaxis are RCM, dextran, and some NSAIDs (20, 122, 123).

### Non-Immunologic Anaphylaxis

This type of anaphylaxis does not involve the activation of the immune system, rather the direct stimulation of mast cell degranulation, as has been shown for some drugs (104). This process can be mediated through the MAS-related G protein-coupled receptor-X2 (MRGPRX2) (102–104). The interaction of certain drugs with this mast cell receptor can induce the release of histamine, β-hexosaminidase, TNFα, and PGD2 among others, potentially leading to non-allergic anaphylactic reactions. Medications such as quinolones, opioids, vancomycin, RCM, dextrans, and NMBA have been found to directly stimulate mast cells (104, 124). Whether certain factors may predispose individuals to this type of anaphylaxis needs further research.

## Diagnosis of Anaphylaxis and Identification of the Culprit Drug

**Figure 2:**
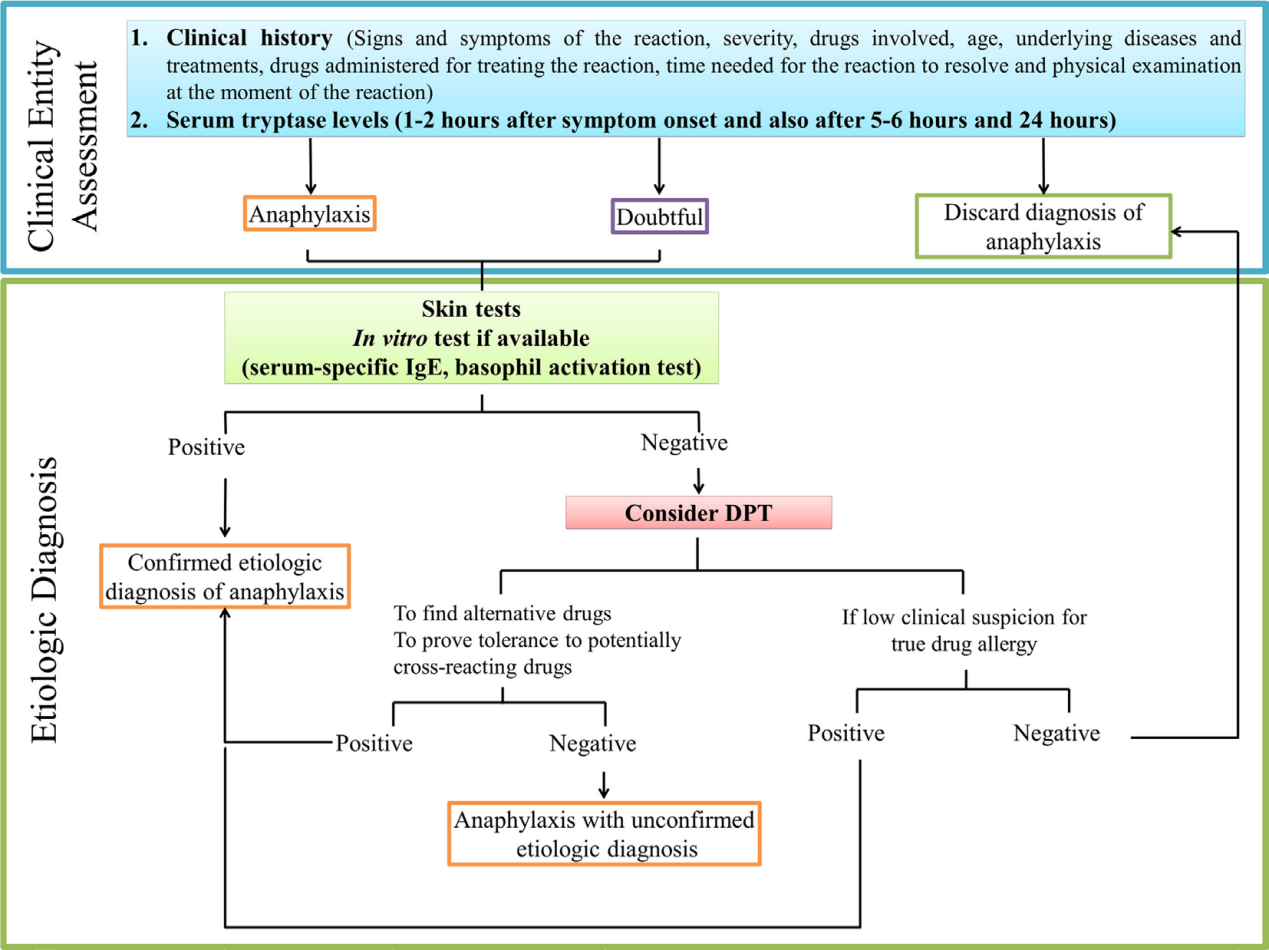
**Recommended practice flowchart for allergy diagnostic work-up in drug-induced anaphylaxis**.

The diagnosis of anaphylaxis is based on a thorough examination of patient history and physical evaluation (125). It is important to evaluate various aspects: clinical signs and symptoms of the reaction, grade of severity, drugs administered for treating the reaction, the time needed for the reaction to resolve, age, underlying diseases, and ongoing treatments, such as beta-blockers and angiotensin-converting enzyme inhibitors, and all possible drugs involved in the episode. An accurate identification of the responsible agent is crucial for avoiding anaphylaxis in future treatments (126). The temporal relation of anaphylaxis after the intake of the drug should be ascertained, as most reactions occur within minutes to hours after exposure. However, different drugs are often taken simultaneously, so clinical history is often inconclusive, in which case the work-up of a suspected drug-induced anaphylaxis should also include skin tests, when available and well validated, and in some cases, although not recommended, drug provocation tests (DPTs) (126). *In vitro* tests can complement the diagnosis confirming clinical suspicions of a severe systemic reaction and avoiding the need to conduct DPTs, potentially saving the patient from suffering another reaction. Moreover, they may help to identify the culprit drug and the underlying mechanism (127). We provide a flowchart for diagnosing drug-induced anaphylaxis in Figure 2.

### *In Vivo* Diagnosis

To assess IgE-mediated anaphylaxis, skin testing including skin prick tests (SPT) and intradermal testing (IDT) should be performed. For drug-induced anaphylaxis, SPT are typically performed with the undiluted drug. If negative, IDT is performed sequentially with increasing concentrations of the drug, due to the potential risk of inducing systemic symptoms (128). A positive skin test response is defined by the size of the wheal, which should be 3 mm or greater than that of the negative control (129). Testing should be performed as soon as possible to avoid loss of test sensitivity over time reported for IgE-mediated reactions to drugs (130, 131); although it should not be performed less than 6 weeks after the episode, to avoid any possible refractory period in which testing may give a false negative (24) The rate of negativization depends on the drug, ranging from 60% after 6 months for dipyrone (131) to 47% within 4 years for NBMAs (132).

For most drugs, a negative skin test does not rule out allergy. Therefore, DPT is generally accepted as the gold standard; however, it is not recommended in anaphylaxis due to the high risk of inducing another reaction. It is primarily indicated for patients where clinical suspicion is low, and for patients where it is essential that alternatives to an implicated drug are found (24). It can also be recommended for assessing tolerance to potentially cross-reactive drugs (24). It must be performed under expert supervision, where resuscitation facilities are available and early signs of disorders arising from DPT can be detected (133). Although the traditional drug challenge consists of stepwise graduations, one-step and two-step test dose strategies have been suggested recently (134). Nevertheless, since crucial cofactors might be absent during the procedure, its sensitivity may be not optimal.

### *In Vitro* Diagnosis

Mast cell mediator release can be analyzed immediately after symptom onset and can be considered useful for diagnosis. Tryptase is among the early mediators released by mast cells during an acute allergic reaction, often showing elevated serum levels (>11.5 ng/mL) in anaphylaxis. The measure of total serum tryptase is the most widely used laboratory test to confirm anaphylaxis. As its levels peak 1–2 h after symptom onset and normalize after 5–6 h (101), the optimal timing for drawing a tryptase concentration is 1–2 h after the event (24). However, a normal tryptase level does not rule out anaphylaxis, and values obtained at the time of the event should always be compared with a recent baseline serum tryptase (135, 136). Indeed, a relative increase greater than 135% of the baseline value (even below 11.4 ng/mL) has been suggested to improve diagnosis (137).

Histamine is the first mediator released by mast cells; any elevation in plasma or urine is consistent with anaphylaxis. However, normal levels do not exclude diagnosis and, like tryptase, the acute level must be compared with baseline (127). However, plasma histamine has short half-life (20 min), which limits the utility of this measurement in the clinical setting (101, 138). An indirect method for the determination of histamine consists of measurement of its metabolites, *N*-methylhistamine or *N*-methylimidazoleacetic acid, in urine. These appear within 30–60 min of the event and stay detectable for a 24-h period (98, 139, 140).

In addition, levels of chymase, mast cell carboxypeptidase A3, PFA, and other mast cell products may prove to be useful as biomarkers for anaphylaxis (141).

When immunologic mechanisms are involved in the reaction, additional laboratory assays, such as serum-sIgE quantification or the basophil activation test, can be useful to confirm the culprit drug. Immunoassays for drug-sIgE determination using ImmunoCAP are available for a handful of drugs, including five beta-lactams, NMBAs, chlorhexidine, and a few other biological agents (127). Although immunoCAP is the most widely used method, custom-made radioimmunoassays can also be used for a wider variety of drugs including quinolones and other beta-lactams (127). The basophil activation test, which can be performed with any suspected drug, measures the activation of basophils after stimulation and is suitable for both IgE-mediated and non-IgE-mediated hypersensitivity (24).

## Management

Adrenaline is the first-line treatment for anaphylaxis and should be administered as soon as possible by intramuscular injection into the middle of the outer thigh (142). The patient may require the repeated administration of adrenaline at 5-min intervals if improvement is not observed or symptoms reoccur. Following adrenaline treatment, the trigger should be removed if possible, for example, stopping i.v. medication. The administration of other drugs such as corticosteroids and beta-2 agonists may reduce other features of anaphylaxis and the risk of biphasic and protracted reactions (143, 144). Parenteral administration of glucagon may be useful for treating patients who are unresponsive to adrenaline, particularly in those taking beta-blockers (145).

## Conclusion

Drug-induced anaphylaxis is a potentially life-threatening reaction that appears to be increasing in both prevalence and incidence, likely due in part to the introduction of new medications. An accurate and prompt diagnosis is necessary to a correct management of this acute reaction, and the identification of the culprit drug is crucial to avoid new future reactions. Further research about mechanisms and risk factors is needed to try to prevent the development of this reaction and to orient therapeutic approaches to patient, based on the culprit drug and the clinical reactions, which should target the underlying specific mechanisms.

## Author Contributions

All authors have made substantial intellectual contributions to the preparation of the manuscript and approved it for publication.

## Conflict of Interest Statement

The authors declare that the research was conducted in the absence of any commercial or financial relationships that could be construed as a potential conflict of interest.
